# Nursing Roles and Responsibilities in Outpatient Bronchiectasis Care: A Scoping Review

**DOI:** 10.3390/nursrep16070242

**Published:** 2026-07-14

**Authors:** Keirran Hiscock, Rebecca Miriam Jedwab

**Affiliations:** 1 Adults Cystic Fibrosis and Respiratory Research Department, The Prince Charles Hospital, Metro North, Chermside, Brisbane, QLD 4032, Australia; 2 Nursing and Midwifery Informatics, Department of Clinical Informatics, Monash Health, Clayton, VIC 3168, Australia; rebecca.jedwab@monashhealth.org

**Keywords:** bronchiectasis, health workforce, nurses, outpatient, respiratory tract diseases

## Abstract

**Background:** Bronchiectasis is a chronic respiratory condition characterised by irreversible airway dilation and recurrent infections, resulting in significant symptom burden and frequent healthcare utilisation. Although nurses are central to chronic respiratory disease management, their specific roles and responsibilities in outpatient bronchiectasis care remain poorly defined. Understanding these roles is imperative to support workforce planning, optimise multi-disciplinary collaboration, and improve patient outcomes. **Aim:** This scoping review aimed to map and synthesise existing evidence on the roles and responsibilities of nurses involved in the outpatient management of adults with non-cystic fibrosis bronchiectasis. **Methods:** A scoping review was conducted. Six databases were systematically searched (MEDLINE, CINAHL Complete, Central, Web of Science, and ProQuest Dissertations and Theses Citation Index). Records describing nursing roles, responsibilities, or models of care within outpatient bronchiectasis settings were included (any design). Data was analysed descriptively and thematically. **Results:** Five studies and two international clinical practice guidelines published between 2002 and 2025 were included. Nurses were shown to play roles across five key domains: 1. clinical assessment and monitoring, 2. self-management support and patient education, 3. care co-ordination and multi-disciplinary collaboration, 4. patient advocacy and communication, and 5. leadership and service development. Evidence on measurable outcomes and standardised role definitions remains limited. **Conclusions:** This review mapped five domains within which nurses may contribute to outpatient bronchiectasis care; however, most identified roles and responsibilities were derived from multi-disciplinary recommendations rather than explicit descriptions of nursing practice. Further research is required to better define nursing roles and responsibilities and evaluate nurse-led models of care in bronchiectasis outpatient care settings.

## 1. Introduction

Bronchiectasis is a chronic respiratory disorder marked by the abnormal and irreversible widening of the bronchial airways, resulting in persistent productive cough, recurrent respiratory infections, and significant symptom burden [1]. Diagnosis relies on high-resolution computed tomography (HRCT), in conjunction with clinical assessment [2]. While cystic fibrosis (CF)-related bronchiectasis has long been studied, non-cystic fibrosis bronchiectasis (NCFB) is increasingly recognised as a distinct and growing health burden, particularly in outpatient settings where long-term monitoring and patient education are required [3].

### 1.1. Bronchiectasis Prevalence and Epidemiology in Australia

In the Australian context, the incidence of bronchiectasis appears to be rising, particularly among older adults and Aboriginal and Torres Strait Islander (First Nations) populations. Data from the Australian Institute of Health and Welfare [4] reported nearly 18,000 bronchiectasis-related hospitalisations in 2017–2018, with the highest rates among those aged 75 and older. Aboriginal and Torres Strait Islander people experience disproportionately higher rates of bronchiectasis, with earlier onset and more severe clinical outcomes [5]. Research from the Northern Territory has shown that a significant number of Indigenous children develop radiologically confirmed bronchiectasis following recurrent respiratory infections in early childhood [2,6].

### 1.2. Bronchiectasis Management and Multi-Disciplinary Care

The management of bronchiectasis is multifaceted and requires collaboration across healthcare disciplines, in both inpatient and outpatient settings. Core treatment components include airway clearance techniques, antimicrobial therapy, management of inflammation, physical activity and regular microbial surveillance, and symptom monitoring. As with other respiratory disorders, identifying and treating the underlying cause where possible is a key part of clinical care [1]. Nurses, physiotherapists, respiratory physicians, and pharmacists frequently work together to tailor patient-centred care, particularly in outpatient settings, to improve health outcomes and reduce exacerbation rates and hospital admissions [7].

### 1.3. The Role of Nurses in Chronic Respiratory Disease Management

Nurses make important contributions to chronic disease management through care co-ordination, patient education, continuity of care, and support for self-management [8], with nurse-led chronic disease services helping reduce hospitalisations and improve patient satisfaction [9]. Supporting the broader role of respiratory nurses in long-term respiratory disease management, nurse-led interventions have demonstrated benefits in COPD management, quality of life and emotional state measures, pulmonary outcomes, and physical capacity [10]. In the care of patients with bronchiectasis, the treatable traits approach highlights a range of outpatient and community-based interventions that may be delivered by nurses or other allied health professionals. However, bronchiectasis-specific nursing roles and responsibilities remain poorly defined, and there is a significant gap in the literature regarding nursing competencies, scope of practice, and models of care for this patient population [11,12,13].

Studies in asthma and COPD have demonstrated that nurse-led or nurse-co-ordinated care improves treatment adherence, symptom control, and quality of life [10,14]. Although evidence for bronchiectasis-specific nursing models is limited, the complexity and chronicity of the condition suggests a strong rationale for clearly defined nursing roles.

### 1.4. Outpatient Nursing Models in Respiratory Care

Nurse-led clinics in chronic respiratory conditions such as COPD and asthma have shown benefits including reduced hospitalisations and improved patient satisfaction [10]. These models typically focus on structured care plans, patient education, and timely follow-up. Despite the growing prevalence and complexity of bronchiectasis, nursing roles in outpatient bronchiectasis care are inconsistently defined across clinical settings. Existing reviews on bronchiectasis have often concentrated on medical management, pharmacologic interventions, or multi-disciplinary care broadly, without delineating the specific contributions of nurses [7,15]. To date, there is a gap in understanding and defining nursing roles and responsibilities in bronchiectasis outpatient clinics.

### 1.5. Aim

The aim of this review was to explore and map the available literature on the roles and responsibilities of nurses in the management of patients with NCFB within outpatient clinics.

## 2. Methods and Methodology

### 2.1. Design and Rationale

A scoping review methodology was selected to explore the roles and responsibilities of nurses within outpatient clinics. This decision was informed by the need to explore a range of previous studies for suitability, and the limited availability of evidence specifically addressing the topic [16,17]. Unlike systematic reviews, which focus on synthesising evidence from a narrowly defined set of studies, scoping reviews are particularly well-suited for mapping key concepts, identifying gaps in knowledge, and including a broader range of evidence sources [18]. A scoping review offers an appropriate and rigorous framework to address the research aim and inform future investigations and practice development in this area. A protocol was developed and prospectively registered on the Open Science Framework (https://doi.org/10.17605/OSF.IO/Y5TQS) in July 2025 for this scoping review.

Outpatient clinics were selected as the context of interest because bronchiectasis is a chronic condition requiring long-term monitoring, multi-disciplinary management, patient education, and exacerbation prevention, all of which are predominantly delivered within outpatient care settings. The experience of the first author (a nurse who works in the outpatient respiratory care setting) and gaps in evidence related to nurses’ roles and responsibilities in that setting, provided the rationale for examining, mapping and comparing nurses’ roles and responsibilities in outpatient care.

### 2.2. Defining and Aligning the Research Question and Review Aim

The research question for this scoping review was created with the Population–Concept–Context (PCC) framework, which assists in formulating a research question by focusing on the key themes [19]. Therefore, this review aimed to explore and map publications that related to: NCFB or mixed respiratory cohorts with increased relevance to NCFB (population); the reporting or describing of nursing roles, responsibilities or scope of practice (concept); and the outpatient setting (context).

### 2.3. Eligibility Criteria

Eligibility criteria were aligned with the PCC framework and research question. Any included sources in the review required a cohort with a diagnosis of non-cystic fibrosis bronchiectasis (NCFB) or another chronic respiratory condition with distinct data relevant to NCFB. Furthermore, those required the reporting or description of nursing roles, responsibilities, or scope of practice within the outpatient setting. Peer-reviewed literature and grey literature including protocols, clinical practice guidelines, thesis, and expert opinion papers were included to extend the search (included in database searches and manually screened references).

NCFB records that had no reference to nursing roles and responsibilities, or where nurses were only mentioned incidentally without substantive detail, were excluded. Broader bronchiectasis publications were excluded where they discussed interventions or models of care that could be delivered by nurses or allied health professionals but did not provide distinct information on nursing roles, responsibilities, or scope of practice. Conference abstracts lacking detailed methodology or results were also excluded. Papers about paediatric patients were excluded and non-English language records were excluded due to a lack of funding available for transcription. Any published works that were more than 25 years old (from date of searching) were excluded due to wanting to capture the most up-to-date clinical practice for NCFB patients. As per scoping review methodology, no quality appraisal was undertaken, and therefore no records were excluded based on quality [20].

Table 1 presents the search components developed for this study, and the inclusion and exclusion criteria related to each concept.

### 2.4. Study Searching

A comprehensive and systematic search strategy was developed in collaboration with an advanced health librarian to identify studies relevant to bronchiectasis and the role of nursing. The search was conducted on 9 April 2025, and repeated on 1 May 2026, across six major databases: MEDLINE (Ovid), CINAHL Complete (EBSCO), EMBASE (Elsevier), CENTRAL (Cochrane), Web of Science Core Collection, ProQuest Dissertations and Theses Citation Index. The search was repeated to ensure all eligible recent publications were screened. Search terms included both controlled vocabulary (e.g., MeSH and Emtree terms) and free-text keywords related to bronchiectasis, Kartagener syndrome, ciliary dyskinesia, and bronchial dilation, as well as terms associated with nursing roles, nursing staff, and nursing practice. Boolean operators and proximity functions were applied to refine the search and enhance sensitivity. Each database-specific-strategy was tailored to its indexing system and search capabilities. The complete search string for Medline (Ovid is available in Supplementary Material File S1).

### 2.5. Study Selection

All retrieved records were imported into Covidence software for screening and data management. Duplicate entries were automatically removed. Title and abstract screening, followed by full-text review, was conducted independently at each stage by the research team (KL and RMJ), one with academic expertise (nursing) and the other with advanced clinical experience in respiratory nursing. A third reviewer (experienced nursing academic) was available to resolve discrepancies, although this person was not required at either stage. Records that did not meet inclusion criteria at either stage (detailed in Table 1) were removed.

The reference lists of the records included for screening were examined for any other related publications. International clinical practice guidelines that included information on the management of bronchiectasis patients by nurses were manually included for screening at this stage.

### 2.6. Data Extraction and Synthesis

The data extraction table was developed per JBI guidance for scoping reviews and piloted by the research team before commencing data extraction [18]. The table included information relating to nursing activities, responsibilities, scope of practice, service delivery and reported outcomes. Data extraction was undertaken independently by both reviewers and completed in Microsoft Excel to ensure consistency and accuracy.

An inductive thematic synthesis approach was subsequently done as per the JBI recommendations for narrative synthesis of data [18]. Both reviewers independently reviewed the extracted data and identified recurring concepts relating to nursing roles and responsibilities. These concepts were recorded within the data extraction spreadsheet and grouped into preliminary categories. Following completion of data extraction, the reviewers met to compare findings and discuss areas of similarity and difference. Through an iterative consensus process, categories that demonstrated conceptual overlap were grouped into broader thematic domains. Themes were identified that reflected the most consistently reported nursing responsibilities across the included publications. Any differences in interpretation were resolved through discussion and agreement between reviewers.

### 2.7. Review Reporting

The reporting of this review adhered to the Preferred Reporting Items for Systematic Reviews and Meta-Analyses extension for Scoping Reviews (PRISMA-ScR) checklist [21].

### 2.8. Ethical Approval

Ethical approval was not required for this scoping review.

## 3. Results

**Figure 1:**
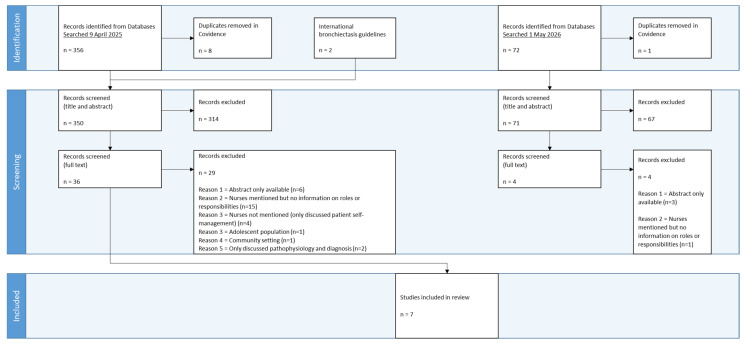
PRISMA flow diagram.

In total, 428 sources were imported into Covidence from the six databases. Nine duplicates were removed, and two international clinical practice guidelines were manually added, leaving 421 screened at title and abstract level. After title and abstract screening, 40 sources were reviewed at the full-text level. After full-text screening, seven were included in this review. The PRISMA flow diagram includes the number of sources screened, included or excluded at each stage, as well as the reasons for exclusion at full-text level, and is included below (Figure 1).

The seven included publications included a mix of study designs, including one randomised controlled trial [22], a systematic review [23], two discussion/commentary papers [24,25], a practice-based review [26] and two international clinical practice guidelines [1,27]. The systematic review by Lawton et al. (2018) [23] itself only included one study, which was also captured in this review as one of the seven included publications [22] and the topic of the editorial by Rafferty & Elborn (2002) [25]. Six publications were from England [1,22,23,24,25,27] and one was from Spain [26].

Across the published works, nurses were consistently described as having essential roles in supporting the ongoing management of patients with NCFB, monitoring their clinical status, and contributing to multi-disciplinary care. The design and focus of each of the included publications as well as their key findings, nursing roles (including the source of this information), and identified impact of nursing on patient outcomes or service delivery is presented in Table 2.

Analysis of the five publications and two international clinical practice guidelines identified five key themes representing the core responsibilities of nurses in bronchiectasis outpatient clinic settings. These include: 1. clinical assessment and monitoring; 2. self-management support and patient education; 3. care co-ordination and multi-disciplinary collaboration; 4. patient advocacy and communication; and 5. leadership and service development. Importantly, the five themes identified in this review were supported by different types of evidence. Direct descriptions of nursing practice were primarily derived from Sharples et al. (2002) [22], Peres (2009) [24], and Cosio et al. (2025) [26], which explicitly described nursing activities, responsibilities, or nurse-led models of care. In contrast, several additional responsibilities were identified through synthesis of multi-disciplinary clinical practice guidelines [1,27], where recommendations were directed to the healthcare team rather than nursing specifically. Consequently, some of the nursing functions presented in this review reflect explicit descriptions of nursing practice, whereas others represent the authors’ interpretation of how multi-disciplinary recommendations may be enacted within outpatient bronchiectasis services. These findings should therefore be interpreted as a conceptual mapping of nursing roles rather than a definitive description of evidence-based nursing competencies. The five themes are discussed below and summarised in Table 3.

### 3.1. Clinical Assessment and Monitoring

Clinical assessment and ongoing monitoring emerged as core nursing responsibilities in outpatient bronchiectasis care. Across the included literature, nurses were responsible for conducting or co-ordinating structured clinical reviews, monitoring symptoms and treatment adherence, and identifying early signs of deterioration. The British Thoracic Society (BTS) guidelines recommend routine monitoring, structured annual reviews, and regular assessment of lung function, exacerbation frequency, sputum microbiology, and treatment tolerance [27]. Although these recommendations are not discipline-specific, they describe clinical processes that are routinely performed through nursing assessment and follow-up in outpatient respiratory services. The European Respiratory Society (ERS) guidelines similarly emphasise severity assessment using validated tools, such as the Bronchiectasis Severity Index, alongside ongoing evaluation of treatable traits and monitoring of adherence to long-term therapies [1]. In the randomised controlled crossover trial by Sharples et al. (2002), nurse practitioners conducted comprehensive assessments equivalent to respiratory physicians, including evaluating symptoms, lung function, and treatment adherence [22]. These assessments formed the basis for tailored management plans and facilitated continuity of care within the outpatient setting. Similarly, Cosio et al. (2025) described nurses’ involvement in structured respiratory assessments as part of a broadened multi-disciplinary clinic, reinforcing their contribution to ongoing clinical surveillance [26]. Although these recommendations are not discipline-specific, their inclusion within this theme represents an interpretive synthesis by the review authors, rather than explicit evidence that these activities are routinely performed by nurses in bronchiectasis services.

### 3.2. Self-Management Support and Patient Education

A strong correlation across the included papers was apparent in the educational and supportive roles of nurses in helping patients manage their condition. Peres (2009) highlighted the central role nurses play in promoting patient self-management through education about treatment adherence, symptom recognition, and lifestyle medication [24]. Similarly, in Sharples et al. (2002) [22], and therefore mentioned in Lawton et al. (2018) [23], it was found that nurse-led care models contributed to improved patient understanding and engagement with their treatment. Nurses provided individualised education and support to assist patients in recognising the early signs of exacerbation and maintaining their adherence to their care plans, which are important components of long-term disease management. The ERS guidelines place strong emphasis on patient education, supported self-management, early recognition of exacerbations, and adherence to long-term treatment strategies [1]. The BTS guidelines similarly advocate for patient education on airway clearance techniques, exercise, and the use of personalised self-management plans [27]. While these guidelines do not specify professional roles, the delivery and reinforcement of education described are consistent with routine nursing responsibilities in outpatient clinics. These recommendations support the educational and supportive roles identified across the included publications, where nurses assisted in delivering individualised education and reinforcing self-management behaviours within bronchiectasis outpatient clinics. The nursing contribution to this theme is supported by direct descriptions of practice in Peres (2009) [24] and Sharples et al. (2002) [22], while the broader guideline recommendations required interpretation regarding potential nursing involvement.

### 3.3. Care Co-Ordination and Multi-Disciplinary Collaboration

Effective co-ordination of care was identified as a defining aspect of the nursing role in bronchiectasis outpatient settings. Nurses often acted as the central co-ordinating clinician between patients and the broader multi-disciplinary team, including physicians, physiotherapists, and other allied health professionals. Cosio et al. (2025) explained how respiratory specialist nurses were integrated within a treatable traits model, facilitating communication across disciplines and ensuring that care was individualised and consistent [26]. Peres (2009) also described the importance of nurse’ co-ordination efforts in managing referrals, monitoring treatment outcomes, and liaising with community health services [24]. This co-ordination was shown to assist in continuity and reduce gaps in care. Both the BTS and ERS guidelines strongly endorse multi-disciplinary approaches to bronchiectasis management, particularly for patients with moderate to severe disease or frequent exacerbations [1,27]. Although not discipline-specific, these recommendations reflect care processes that are commonly enacted through nursing co-ordination roles in outpatient bronchiectasis clinics. Direct evidence for this role was available from Peres (2009) [24] and Cosio et al. (2025) [26], whereas guideline recommendations informed the broader conceptualisation of nursing involvement in multi-disciplinary care.

### 3.4. Patient Advocacy and Communication

Nurses were described as key advocates for patients, ensuring their perspectives and preferences were recognised in clinical decision-making. Across the sources, nurses played an important role in communicating between patients and the healthcare team. Peres (2009) [24] and Rafferty & Elborn (2002) [25] (and therefore Sharples et al., 2002 [22]) emphasised that the relationships between the nurse specialists and patients, building trust and effective communication, were important in supporting patient wellbeing. These sources identified advocacy in the patients’ interests and in promoting equitable access to services and supporting patients in navigating complex healthcare systems. BTS guidelines emphasise shared decision-making and consideration of patient preferences, particularly in relation to airway clearance techniques and long-term treatment choices [27]. These principles align closely with established nursing practice in chronic disease management and reinforce the advocacy and communication roles described across the included publications. This theme derived predominantly from nursing-focused publications, rather than multi-disciplinary guideline recommendations.

### 3.5. Leadership and Service Development

Leadership was noted as a developing but less consistently mentioned theme within the literature. Sharples et al. (2002) reported comparable clinical outcomes between nurse practitioner-led and physician-led outpatient care [22]. However, the study also identified higher resource utilisation within the nurse-led model, and the findings should be interpreted cautiously given the age of the study and the absence of subsequent confirmatory research. Cosio et al. (2025) also described how nurses contributed to service innovation through participation in the design and delivery of multi-disciplinary respiratory units [26]. The ERS guideline calls for structured implementation of evidence-based interventions, including airway clearance education and pulmonary rehabilitation, alongside systems to support adherence and monitor outcomes [1]. Similarly, the BTS guidelines recommend specialist bronchiectasis clinics with appropriate governance structures for patients with complex disease [27]. While these guidelines do not explicitly define nursing leadership roles, the activities they recommend, such as education delivery, pathway implementation, and outcome monitoring, align with advanced and specialist nursing responsibilities. While the evidence remains limited, these findings highlight opportunities for nurses to lead quality improvement initiatives, contribute to clinical governance, and shape the development of future outpatient bronchiectasis care models. Of all themes identified, leadership and service development relied most heavily on indirect evidence and conceptual interpretation of service-level recommendations.

## 4. Discussion

This scoping review helps fill a gap in the literature by exploring and mapping available evidence on the roles and responsibilities of nurses within bronchiectasis outpatient care. Five key themes were identified: clinical assessment and monitoring, self-management support and patient education, care co-ordination and multi-disciplinary collaboration, patient advocacy and communication, and leadership and service development. These themes provide examples of established nursing roles and responsibilities within bronchiectasis care; however, only one of the seven included publications directly evaluated nurse-led outpatient bronchiectasis care. Several identified nursing responsibilities came from multi-disciplinary clinical practice guidelines or descriptive publications rather than direct evaluation of nursing practice. Consequently, by mapping potential nursing roles and responsibilities in bronchiectasis outpatient settings, the findings from this review should be considered conceptual and exploratory.

There are several notable gaps in the examined literature. First, there is no standardised framework defining nursing roles and competencies specific to bronchiectasis, and second, evidence evaluating the effectiveness or outcomes of nursing-led interventions remains limited. Third, most existing publications are descriptive or opinion-based, with few measurable evaluations. There was large overlap in the included publications of this review, where the Sharples et al. (2002) [22] study was also included in the systematic review [23] and Rafferty & Elborn’s editorial (2002) [25]. Following the guidance for conducting a scoping review meant each publication was included in our analysis, although this emphasises the limited published evidence on this topic. Finally, little research has explored leadership or governance functions within respiratory nursing. Addressing these gaps through targeted research will help strengthen nursing workforce planning, inform policy, and improve multi-disciplinary collaboration.

The limited findings from this review mirror evidence from other chronic respiratory nursing areas, particularly COPD and asthma. Nurse-led COPD clinics, for example, have demonstrated improved symptom control, reduced hospital admissions, and increased patient satisfaction through structured follow-up, self-management education, and early intervention [10]. Similarly, asthma nurse specialist models promote self-management through education and regular monitoring. The themes developed in this review align closely with established Advance Practice Nursing (APN) frameworks, which identify clinical practice, leadership, education, research and collaboration as core dimensions of advanced practice nursing [14]. Within bronchiectasis outpatient care, the themes of clinical assessment and monitoring reflect advanced clinical practice, while self-management support and patient education align with the education function of APN roles. Similarly, care co-ordination and multi-disciplinary collaboration reflect the systems-level and collaborative aspects of advanced practice nursing, whilst leadership and service development correspond to the leadership and quality improvement components of the APN framework. Although evidence specific to bronchiectasis care remains limited, the APN framework provides a useful theoretical foundation for future development of role descriptions and competency standards in this clinical setting.

International clinical practice guidelines that include nurses in the multi-disciplinary management of patients with bronchiectasis represent best practice suggestions rather than professional standards of nursing practice, and are mostly based upon expert consensus rather than robust empirical evidence [1,27]. This highlights an ongoing need for standardised frameworks to articulate nursing roles and evaluate their impact on clinical outcomes within outpatient bronchiectasis services. As well as standardised frameworks, standardised nursing role descriptions may improve care delivery, facilitate communication with multi-disciplinary teams, and inform policy and workforce planning. Defining and formalising nursing roles within bronchiectasis outpatient care may also support continuity of care, strengthen multi-disciplinary communication, and ensure consistent quality standards. Evidence from nurse-led chronic disease clinics shows that structured models improve adherence, patient confidence, and self-management capacity [10]. Integrating similar models into bronchiectasis clinics could improve patient outcomes, reduce exacerbation-related admissions and improve resource use. Formal frameworks may also help elevate nursing leadership, ensuring nurses contribute meaningfully to clinical governance and service innovation.

Future research should focus on adapting existing respiratory nursing models, such as those for COPD, asthma and advanced practice nursing, to outpatient bronchiectasis care. Findings also align with principles of the Chronic Care Model, which emphasises proactive follow-up, self-management support, multi-disciplinary collaboration and co-ordinated long-term care [28]. Bronchiectasis shares many characteristics with other chronic respiratory diseases, including the need for ongoing monitoring, treatment adherence support and early intervention during disease deterioration. The findings related to nursing roles therefore appear consistent with established chronic disease management frameworks. While evidence from COPD and asthma nursing models provides useful insights, direct transferability is limited at this time. Bronchiectasis differs in disease heterogeneity, microbiological surveillance requirements, airway clearance burden, and the frequent need for sputum-guided management. Consequently, although education, self-management support, care co-ordination, and routine monitoring are likely transferable components, disease-specific competencies relating to airway microbiology, exacerbation assessment, and bronchiectasis severity stratification may require additional training and role development.

A key contribution of this review is the development of a conceptual framework describing five domains within which nurses may contribute to outpatient bronchiectasis care. Importantly, this framework should not be interpreted as an evidence-based competency framework or professional standard for bronchiectasis nursing practice. Rather, it represents a synthesis of the limited available literature, combining explicitly described nursing activities with nursing functions inferred from multi-disciplinary recommendations. The framework may therefore provide a foundation for future role development, research, workforce planning, and evaluation of nursing models of care, while highlighting areas where empirical evidence remains absent.

## 5. Limitations

This scoping review has several limitations to note. The inclusion of only English language papers may have led to the incidental exclusion of relevant works. The specificity of our criteria and search terms may have been a limitation in the number of publications identified for relevance (i.e., bronchiectasis-specific, not other chronic respiratory diseases); however, the subject warranted specificity, and the limited number and nature of included studies further supports this justification. Six out of the seven publications were from England; therefore, the generalisability of review findings may be impacted by their setting, including healthcare models, funding, and perceptions of multi-disciplinary care teams in outpatient settings. However, the exploration of the potential impact of funding models on nurses and/or bronchiectasis patients from England and Spain is beyond the scope of this review. It is also important to note the considerable overlap between the included publications, and the amount of time that has passed since their publication (ranging from 2002 to 2025).

## 6. Conclusions

This scoping review identified five domains within which nurses may contribute to outpatient bronchiectasis care: clinical assessment and monitoring, self-management support and patient education, care co-ordination and multi-disciplinary collaboration, patient advocacy and communication, and leadership and service development. However, only one included study directly evaluated nurse-led outpatient bronchiectasis care, and many identified responsibilities were derived from multi-disciplinary recommendations rather than explicit descriptions of nursing practice. Consequently, the principal contribution of this review is the proposal of a conceptual framework to guide future research, role development, and service design rather than the establishment of evidence-based nursing competencies. Further empirical research is required to define nursing roles, evaluate nurse-led models of care, and determine the impact of nursing interventions on patient and service outcomes.

## Figures and Tables

**Table 1:** Search components and inclusion and exclusion criteria.

Components	Inclusion	Exclusion
Population	Cohort with a diagnosis of NCFB (or mixed respiratory cohort with distinct NCFB data) Adult patients (>18 years old)	Paediatric populations (<18 years old)
Reporting or describing nursing roles, responsibilities or scope of practice	Reporting or description of nursing roles, responsibilities, or scope of practice	No reference to nursing roles and responsibilities Nurses only mentioned incidentally without substantive detail (e.g., clinic nurse present)
Outpatient setting	Outpatient settings including clinics	Home care
Other search limitations	Peer-reviewed literature and grey literature including protocols, guidelines, thesis, and expert opinion papers English language Published 2000 onwards	Conference abstracts lacking detailed methodology or results Non-English language Published prior to 2000

**Table 2:** Study summaries.

Author(s) and Year	Design and Focus	Nature of Evidence	Key Findings	Nursing Roles (or Role Components) Identified	Source of Nursing Role Evidence	Impact on Patient Outcomes and Service Delivery
Chalmers et al. (2025) [1]	International clinical practice guideline using GRADE methodology.	Guideline evidence. Provides international recommendations for adult bronchiectasis management. Not primarily designed to define nursing roles.	Strong recommendations for airway clearance, pulmonary rehab, pharmacological management. This guideline suggests treatable traits practice and severity scoring. This guideline includes direct reference to trained nurses in relation to airway clearance education, but most recommendations are broader multi-disciplinary or service-level recommendations rather than nursing-specific role statements.	Teaching airway clearance techniques by respiratory physiotherapists or trained nurses. Education on self-management plans. Monitoring adherence and technique. Supporting pulmonary rehabilitation programmes.	Mixed: Explicit nursing references plus multi-disciplinary recommendations.	Provides evidence-based clinical practice recommendations to improve symptom control and quality of life, reduce exacerbations, improve proactive management, and reduce hospitalisations and disease progression for patients with bronchiectasis. However, nursing-specific outcomes are not separately evaluated.
Cosio et al. (2025) [26]	Discussion of a nurse-led respiratory unit integrating ‘treatable traits’ framework.	Describes an integrated respiratory care model involving multi-disciplinary practice. Provides contemporary service-based evidence but is not primarily an outcome evaluation of bronchiectasis-specific nursing care.	Highlights the role of respiratory specialist nurses in multi-disciplinary management and personalised care models. Focuses on the integration of nurses into treatable-trait-based management systems.	Specialist respiratory nursing, assessment of treatable traits, co-ordination of care, multi-disciplinary collaboration and health education.	Direct descriptive nursing evidence.	Enhanced personalisation of care and integration of nursing expertise in disease phenotyping and management. Suggests that nursing involvement may enhance personalisation of care and integration of multi-disciplinary expertise. However, bronchiectasis-specific nursing outcomes are not independently measured.
Hill et al. (2019) [27]	UK national guideline based on systematic review and expert consensus.	Guideline evidence. Provides recommendations for adult bronchiectasis management, including monitoring, airway clearance, long-term therapies, pulmonary rehabilitation, and management of deteriorating patients.	Emphasises airway clearance, physiotherapy, long-term antibiotics, and pulmonary rehab; includes detailed practical steps for deteriorating patients. This guideline recommends care processes that may involve nurses in outpatient services, but most recommendations are not assigned specifically to nursing roles.	Education and reinforcement of airway clearance techniques. Support for adherence and self-management plans. Co-ordination of care for deteriorating patients. Monitoring for adverse effects of long-term therapies.	Multi-disciplinary recommendations for nursing practice.	Improved quality of life, reduced exacerbation frequency, structured follow-up improves continuity and safety. However, the guideline does not provide nursing-specific standards or evaluate nursing-led outcomes.
Lawton et al. (2018) [23]	Cochrane systematic review comparing nurse-led versus doctor-led care in bronchiectasis.	Overlapping evidence; the reviewer identified only one eligible study [22], which is also included in this scoping review. Therefore, this source does not represent an independent empirical evidence base.	Only one eligible study met inclusion criteria [22] (incidentally also included as a study in this scoping review). The review concluded that while evidence supports nurse-led care as safe and effective, more research is needed to establish its generalisability.	Nurse-led care co-ordination and monitoring; leadership in outpatient management.	Secondary evidence (overlapping with Sharples et al. (2002) [22]).	Concluded that nurse-led care may be safe and effective, but the evidence base was extremely limited and further research was required to establish generalisability.
Peres (2009) [24]	Practice-based review of bronchiectasis management and nursing responsibilities.	Nursing-focused descriptive source. Provides direct discussion of nursing roles but does not report primary empirical outcome data.	Describes the roles of nurses in patient education adherence support and treatment management. Emphasises interprofessional collaboration and long-term care continuity.	Patient education, adherence support, airway clearance, psychosocial support, co-ordination of multi-disciplinary care.	Direct descriptive nursing evidence.	Improved patient engagement and understanding; strengthened role of nursing in chronic disease management. However, these impacts are described rather than empirically measured.
Rafferty & Elborn (2002) [25]	Editorial discussing nurse-led vs. physician-led outpatient care for bronchiectasis.	Published in relation to the Sharples et al. (2002) trial [22] and should be interpreted as commentary on that study rather than independent evidence.	Supports nurse-led care as a viable alternative to traditional physician-led models, emphasising efficiency.	Leadership in outpatient follow-up, ongoing monitoring, and patient education.	Commentary (overlapping with Sharples et al. (2002) [22]).	Potentially improves continuity and accessibility of care. Supports nurse-led care as a potentially viable model, but conclusions are based on commentary and overlap with the Sharples et al. (2002) trial [22].
Sharples et al. (2002) [22]	Randomised controlled crossover trial comparing nurse practitioner-led versus doctor-led bronchiectasis clinic (n = 62; 1 year in each arm).	This is the only included primary empirical study directly evaluating nurse-led outpatient bronchiectasis care.	The study demonstrated clinical and health-related quality of life outcomes equivalent between nurse-led and doctor-led care. Resource use was significantly higher with nurse-led care.	Assessment, clinical monitoring, patient education, treatment co-ordination, liaison with multi-disciplinary teams.	Direct empirical nursing evidence.	Feasible and safe with comparable clinical outcomes, higher resource utilisation, highlighting the need for structured implementation planning. Findings should be interpreted cautiously due to the age of the study and changes in contemporary bronchiectasis management.

**Table 3:** Summary of key findings.

Theme	Explicit Evidence Related to Nursing Roles	Multi-Disciplinary Evidence Relevant to Nursing Roles	Key Findings and Supporting Studies
1. Clinical assessment and monitoring	Sharples et al. (2002) reported nurse practitioners conducting clinical assessments, symptom review, treatment monitoring and follow-up [22]. Cosio et al. (2025) described respiratory specialist nurse involvement in patient assessment within a multi-disciplinary service model [26].	BTS and ERS guidelines recommend routine monitoring, annual review, exacerbation assessment, severity scoring and adherence monitoring, although these recommendations are directed to the multi-disciplinary team rather than nurses specifically [1,27].	Available evidence suggests nurses may contribute to ongoing assessment and monitoring in outpatient bronchiectasis services, although direct evidence remains limited [1,22,26,27].
2. Self-management support and patient education	Peres (2009) explicitly described nursing responsibilities relating to patient education, adherence support and treatment management [24]. Sharples et al. (2002) incorporated education and support within the nurse-led model [22].	BTS and ERS guidelines advocate multi-disciplinary management involving respiratory physicians, physiotherapists, and other healthcare professionals [1,27].	Although care co-ordination was not extensively evaluated, the available literature suggests nurses frequently function as a link between patients and multi-disciplinary teams [1,22,23,24,27].
3. Care co-ordination and multi-disciplinary collaboration	Peres (2009) highlighted co-ordination of referrals and multi-disciplinary communication [24]. Cosio et al. (2025) described specialist nurses operating within integrated respiratory services [26].	BTS and ERS guidelines advocate multi-disciplinary management involving respiratory physicians, physiotherapists and other healthcare professionals [1,27].	Nurses appear to play an important coordinating role between patients and multi-disciplinary teams, although formal evaluation is lacking [1,24,26,27].
4. Patient advocacy and communication	Peres (2009) highlighted communication and patient support roles [24]. Rafferty and Elborn (2002) discussed the value of nurse-patient relationships and continuity of care [25].	BTS guidelines support shared decision-making and consideration of patient preferences in treatment planning [27].	Advocacy roles were predominantly described in nursing-focused publications and aligned with broader principles of chronic disease management [1,24,25,27].
5. Leadership and service development	Sharples et al. (2002) evaluated a nurse-led outpatient service [22]. Cosio et al. (2025) described specialist nurse involvement in service design and implementation [26]. Rafferty and Elborn (2002) [25] comment on the nurse-led model but this overlaps with the paper by Sharples et al. (2002) [22].	BTS and ERS guidelines support structured specialist services and implementation of evidence-based care processes [1,27].	Limited evidence suggests nurses may contribute to leadership and service development; however, this theme was supported primarily by indirect evidence and requires further investigation [1,22,26,27].

## Data Availability

The original contributions presented in this study are included in the article/Supplementary Material. Further inquiries can be directed to the corresponding author.

## Supplementary Materials

The following supporting information can be downloaded at https://www.mdpi.com/article/10.3390/nursrep16070242/s1. Supplementary Material File S1. Medline (Ovid) search; Supplementary Material File S2. Preferred Reporting Items for Systematic reviews and Meta-Analyses extension for Scoping Reviews (PRISMA-ScR) Checklist.

## Public Involvement Statement

There was no public involvement in any aspect of this research.

## Guidelines and Standards Statement

This manuscript was drafted against the Preferred Reporting Items for Systematic Reviews and Meta-Analyses extension for Scoping Reviews (PRISMA-ScR) checklist.
