# Supplementary material for: Nursing Roles and Responsibilities in Outpatient Bronchiectasis Care: A Scoping Review
Source: Nurs Rep. 2026 Jul 14;16(7):242. doi: 10.3390/nursrep16070242 (PMC13414935; doi:10.3390/nursrep16070242)
Supplement: Supplementary file 1 [file nursrep-16-00242-s001.zip › Bronch scoping review supplementary file 1_10 June 2026.pdf]

Supplementary file 1 - MEDLINE (Ovid) search

S7

S3 AND S6

S6

S4 OR S5

S5

XB nurs* OR CI nurs*

S4

(MH "Nurses+") OR (MH "Nurse's Role") OR (MH "Nursing+") OR (MH "Nursing staff+")

S3

S1 OR S2

S2

XB (bronchiect* OR bronchoect* OR kartagener* OR (ciliary N2 dyskinesia) OR (bronchial* N2 dilat*)) OR CI (bronchiect* OR bronchoect* OR kartagener* OR (ciliary N2 dyskinesia) OR (bronchial* N2 dilat*))

S1

(MH "Bronchiectasis+")

Limiters: English and year 2000 onwards
